# "Right Time, Right Place" Health Communication on Twitter: Value and Accuracy of Location Information

**DOI:** 10.2196/jmir.2121

**Published:** 2012-11-15

**Authors:** Scott H Burton, Kesler W Tanner, Christophe G Giraud-Carrier, Joshua H West, Michael D Barnes

**Affiliations:** ^1^Brigham Young UniversityComputational Health Science Research Group, Department of Computer ScienceProvo, UTUnited States; ^2^Brigham Young UniversityComputational Health Science Research Group, Department of Health ScienceProvo, UTUnited States

**Keywords:** Twitter, GPS Location, Infodemiology, Surveillance, Intervention, Social Media

## Abstract

**Background:**

Twitter provides various types of location data, including exact Global Positioning System (GPS) coordinates, which could be used for infoveillance and infodemiology (ie, the study and monitoring of online health information), health communication, and interventions. Despite its potential, Twitter location information is not well understood or well documented, limiting its public health utility.

**Objective:**

The objective of this study was to document and describe the various types of location information available in Twitter. The different types of location data that can be ascertained from Twitter users are described. This information is key to informing future research on the availability, usability, and limitations of such location data.

**Methods:**

Location data was gathered directly from Twitter using its application programming interface (API). The maximum tweets allowed by Twitter were gathered (1% of the total tweets) over 2 separate weeks in October and November 2011. The final dataset consisted of 23.8 million tweets from 9.5 million unique users. Frequencies for each of the location options were calculated to determine the prevalence of the various location data options by region of the world, time zone, and state within the United States. Data from the US Census Bureau were also compiled to determine population proportions in each state, and Pearson correlation coefficients were used to compare each state’s population with the number of Twitter users who enable the GPS location option.

**Results:**

The GPS location data could be ascertained for 2.02% of tweets and 2.70% of unique users. Using a simple text-matching approach, 17.13% of user profiles in the 4 continental US time zones were able to be used to determine the user’s city and state. Agreement between GPS data and data from the text-matching approach was high (87.69%). Furthermore, there was a significant correlation between the number of Twitter users per state and the 2010 US Census state populations (r ≥ 0.97, P < .001).

**Conclusions:**

Health researchers exploring ways to use Twitter data for disease surveillance should be aware that the majority of tweets are not currently associated with an identifiable geographic location. Location can be identified for approximately 4 times the number of tweets using a straightforward text-matching process compared to using the GPS location information available in Twitter. Given the strong correlation between both data gathering methods, future research may consider using more qualitative approaches with higher yields, such as text mining, to acquire information about Twitter users’ geographical location.

## Introduction

People’s daily use of technology creates “digital breadcrumbs—tiny records of [their] daily experiences” that, when mined and analyzed, can provide insight into health behavior and health outcomes [1]. Traditional behavioral assessments rely on self-report or observation, but increased use of mobile communication devices linked to the Internet and social media applications (apps) are creating unprecedented opportunities for collecting real-time health data and delivering health innovations. For example, mHealth represents a new form of health care delivery and treatment where patients are able to interact with their health care providers through mobile devices—providing additional “breadcrumbs” for studying/mining health behaviors and health outcomes [2].

Although some researchers have expressed concerns about the use of social media in public health [3], an increasing number of researchers welcome the novel opportunities offered by social media to complement (and partially replace in some cases) existing practices in public health and health communication [4-6]. A number of recent studies have demonstrated the value of online information for understanding public health problems and their determinants in areas as diverse as influenza and cholera outbreaks [7,8], tobacco-related issues [9-11], problem drinking [12], dental pain [13], breastfeeding [14], and others [15,16]. This new real-time observation and analysis of user-generated health content in social media has given rise to the terms infoveillance and infodemiology (the study and monitoring of online health information) [17].

Social media connect a wide variety of individuals around many topics and provide a new way for them to share information, reach out, and exchange ideas. As recently editorialized by Ratzan [18], “This change to the way people learn, think, and communicate has revolutionized the context in which health information...needs to be communicated.” Not only is the context different, so is the sheer volume and scale. Millions of individuals worldwide can be reached almost instantaneously with textual, pictorial, and video messages that could alter health behaviors. Additionally, the distribution of social media usage suggests that health disparities may be reduced, and traditionally underrepresented groups and low-income populations may be reached more effectively [19].

As a kind of “listening ear” to the conversations of the world, social media enable health surveillance in completely novel ways. Whereas researchers have relied on questionnaires and focus groups to understand the opinions and behaviors of the public in the past, by using social media they can now observe Internet postings about users’ attitudes and behaviors, many of which can be accessed in real time. These approaches are optimistic because they are typically less expensive and may better reflect the real-life context of behavioral indicators as part of everyday living than traditional assessments of health behaviors. Further, online surveillance enables researchers to study trends as they happen, removing the delay that often arises from designing, administering, and collecting questionnaire-style responses. In addition, by observing users as they interact naturally with one another and their environment, researchers can study true feelings and avoid the Hawthorne effect where the investigator’s presence can cause unintended influence [20]. Thus, these social media channels “are quickly becoming dominant sources of information on emerging diseases” [21].

In addition to using social media for surveillance, these technologies could also be harnessed for health communication and intervention. Although still largely underutilized, social media provide the ability to communicate with people in a completely tailored manner, which has been shown to significantly improve the chances of affecting actual behavior change [22-24]. Furthermore, the real-time nature of the data and the location information of social media provide the opportunity for truly “right time, right place” communication where a person receives the message exactly when and where it is needed. Consider, for example, the possibilities of direct intervention with a potential drunk driver before leaving a party or of a diet reminder reaching a person as they walk into a fast food restaurant. Identifying—and reacting to—health needs in such a timely manner is consistent with Patrick et al [25] and Heron and Smyth [26] who referred to this process as “ecological momentary interventions” or as Intille et al [27] call it, “just-in-time.”

Despite its promise, location in social media is not well understood or well documented. Although proponents of research using social media have pointed to the geolocation information provided by many platforms, such as Twitter, as a means of pinpointing the exact location of users [28], others have cautioned that location information may be underspecified and that location “based on user-identified location or the time zone” could be of questionable quality [3]. The exact Global Positioning System (GPS) coordinates available in some social media platforms could help mitigate this risk because they are direct measures and more difficult to misrepresent. However, unless GPS use is widespread, this does not address the problem of underspecification. Until research is conducted to assess location availability, usability, and the limitations of this data, health practitioners may have limited capacity to observe time- and place-based interventions for determining risks or health conditions.

 The objective of this study is to fill this gap in our understanding of location information in social media, especially as it relates to Twitter. The major contribution of this work is to present the different types of location that can be ascertained from Twitter users and to document the prevalence of each type in an attempt at informing future infoveillance, infodemiology, and health communication research of the availability, usability, and limitations of such location data.

## Methods

Twitter is a social network in which users post status updates, or tweets, that are restricted to 140 characters in length. Users can “follow” others to be notified of their updates, but tweets are also generally available to the public. Because of the public nature of the tweets, users do not have any expectation of privacy, so researchers may openly observe the content. Additionally, Twitter provides a rich application programming interface (API) that enables programmatic searching and retrieval of the data. Twitter users tend to be young and affluent [29]; therefore, one could conclude that they are not representative of entire populations. However, this should not diminish perceptions of Twitter’s utility as a public health tool because it may be an appropriate mechanism for studying attitudes and behaviors of the demographic most represented among its users (ie, young and affluent individuals).

### Location Indicators in Twitter

 Twitter users provide varying degrees of information about their thoughts, attitudes, and behaviors in their profile description and through their tweets. Similarly, they may or may not provide information about their location. When they wish to provide location information, Twitter users have 4 options: (1) exact GPS coordinates associated with a tweet, (2) GPS coordinates of a place (eg, a city or metropolitan region), (3) free-text location information listed in the public profile description, and (4) time zone associated with the user account. Options 1 and 2 are combined into a single setting, the Twitter Location feature, which is disabled by default so that a user must opt-in to use it. Further details about each option and its functionality follow.

 Many users post to Twitter from smartphones or other GPS-enabled devices, and have the ability to broadcast their exact GPS coordinates alongside the text of their tweet. This setting is disabled by default, but when used, this GPS information provides reliable and accurate data about a Twitter user’s location.

 Users posting from their computers and other devices without GPS via the Twitter website can still broadcast their location by providing a GPS “place.” This place is defined by a bounding box of GPS coordinates and often refers to a city or a metropolitan area. This place is inferred by Web browsers, such as Firefox and Google Chrome, and on other browsers through the use of extensions or add-ons. In the case of a GPS-enabled device, this place can be determined directly by the GPS coordinates.

 When users create accounts on Twitter they can fill out a public profile that includes personal information, such as their name, website, bio, picture, and location. Location is an optional text field in which users can enter anything they want. Many users provide their geographical position, such as a city and state/country, but many opt to specify something humorous (eg, “somewhere in my imagination :)” or “a cube world in Minecraft”), sarcastic (eg, “in yhur [bleep!!!] face…” or “Here...obvious!”), or just leave the field blank. The free-text nature of the user-specified location field poses serious challenges. First and most obvious, humorous, sarcastic, and missing entries do not correspond to any identifiable physical location. Second, the entry requires some amount of text processing to correct spelling errors, interpret “textese” and emoticons, and handle abbreviations. Third, the information may be incomplete or ambiguous, such as when a city name is given, but no state or country is provided. Finally, even if the location field can be recognized as a specific location, it is still possible that users chose to provide a location different from where they actually are or that the information is not up-to-date.

Twitter automatically infers a time zone when a user account is created, probably from the local time on the user’s computer or device, and selects it for the user by default. The user can subsequently change this default value, if desired. Although time zones do not denote specific locations, they can still be used to distinguish between major world regions, such as North America and Europe, or the East and West Coasts of the United States. This time zone information could also be helpful in resolving ambiguous city names from profile descriptions.

In addition to these mechanisms supported directly by Twitter, users can also provide location context indirectly in the text of their tweets (eg, “My plane just landed at JFK”) or through third-party applications, such as foursquare (https://foursquare.com/). In some cases, these applications will broadcast GPS coordinates via the standard Twitter mechanisms. In other cases, they may broadcast text or links that would point users elsewhere to see the location. For clarity and to avoid the bias of catering to specific conventions or applications, this study focuses exclusively on the mechanisms supported directly and explicitly by Twitter.

### Data Collection Methodology

The Twitter streaming API provides the ability to receive a portion of the real-time stream of all tweets. This stream can be filtered by certain criteria, such as keywords or a bounding box of GPS coordinates. If no filtering criteria are used (or if the criteria are too general and more than 1% of the tweet stream would be retrieved), the streaming API will return 1% of the total tweets sampled by taking every 100th tweet. As of June 2011 (3 months prior to our data collection), Twitter estimated that approximately 200 million tweets were posted every day [30], resulting in a daily sample of approximately 2 million tweets when using the streaming API with no filter.

Using the Twitter streaming API, we observed the stream of tweets for 2 weeks: October 1-7 and November 7-14, 2011 (approximately 6 hours of the early morning on November 14 were not observed due to a server error). We did not find significant differences between the data of the 2 weeks; therefore, the results presented here are an aggregation of the 2 weeks’ data. By not applying a filter, we received the maximum random sample of 1% of all tweets, yielding a total of 23.8 million tweets posted by 9.5 million unique users. Additionally, because we did not use a filter, our results are not biased by a choice of language or any other artificial means. For each tweet, we recorded the associated location information, both from the tweet itself and from the corresponding user’s profile when applicable.

 Frequencies for each of the location options were calculated to determine the prevalence of the various location data by region of the world, time zone, and state within the United States. Furthermore, data from the US Census Bureau were compiled to determine the proportion of the total United States population living in each state. Pearson correlation coefficients were used to compare states’ populations with the prevalence of Twitter users who enable the GPS location option.

## Results

Table 1 shows the total number of tweets and users, and their distribution over 3 types of location information: exact GPS coordinates (GPS-exact), GPS coordinates of a place (GPS-place), and time zone. In addition, the table shows the percentage of those who had either type of GPS coordinates, which is less than their sum because many users who supplied one also supplied the other. This aggregate value gives a more accurate picture of the amount of reliable (although less specific) location information directly available from tweets.

 There was an average of 2.5 tweets per user. The extremely rapid rate of posting on Twitter (200 million posts per day amounts to more than 2000 tweets per second) and the streaming API’s sampling mechanism (every 100th tweet) mean that it is unlikely that any user is overrepresented or underrepresented. Indeed, the probability that a user could post in exact sync with the streaming API’s sampling is virtually zero. The larger proportions of users who have enabled GPS as opposed to tweets containing GPS information may be explained by the fact that user accounts that run automated applications (ie, bots) are less likely to be GPS-enabled, but may post more frequently and account for more tweets in the sample than regular users. We have not attempted to identify such users here. The remainder of our results are based on unique users identified by their tweets during the 2-week time period.

### Worldwide Distribution

**Figure 1:**
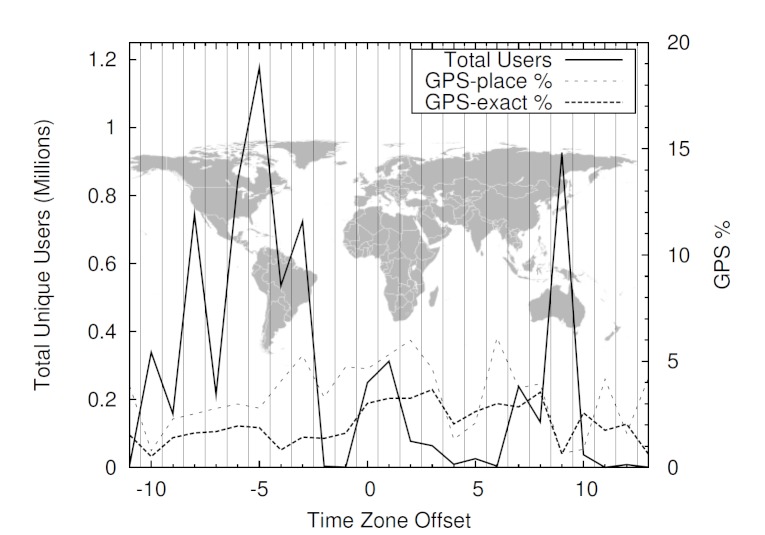
Distribution of Twitter users by time zone (time zones are aligned with longitudes not accounting for deviations based on country borders).

To see whether the number of users and their location information varied across the world, we used the time zone information to overlay these values on a map of the world. The result is displayed in Figure 1, which shows the number of unique users in each time zone who enabled GPS, including the percentage of GPS-exact and GPS-place data. Although the time zones of North and South America have a high number of tweets, European time zones have a higher proportion of tweets that provide GPS information.

### Profile Description Location Information

To parse the free text of the user-supplied information, we used a simple method of looking for text followed by a comma and a state name or abbreviation (ie, “text, state name” or “text, state abbreviation”). This simple parsing method could be improved, yet it provides a useful conservative estimate in its simplicity and efficiency. This method is inherently biased toward English-speaking locations and locations within the United States; therefore, results are shown only for users with time zones listed as one of the US time zones. As a matter of interest, the top 10 pairs parsed (with number of users) are Atlanta, Georgia (10,935); Los Angeles, California (10,244); Chicago, Illinois (8980); Houston, Texas (8147); New York, New York (7804); Washington, District of Columbia (6751); Miami, Florida (5734); Dallas, Texas (5688); Boston, Massachusetts (5562); and Austin, Texas (4678).

**Figure 2:**
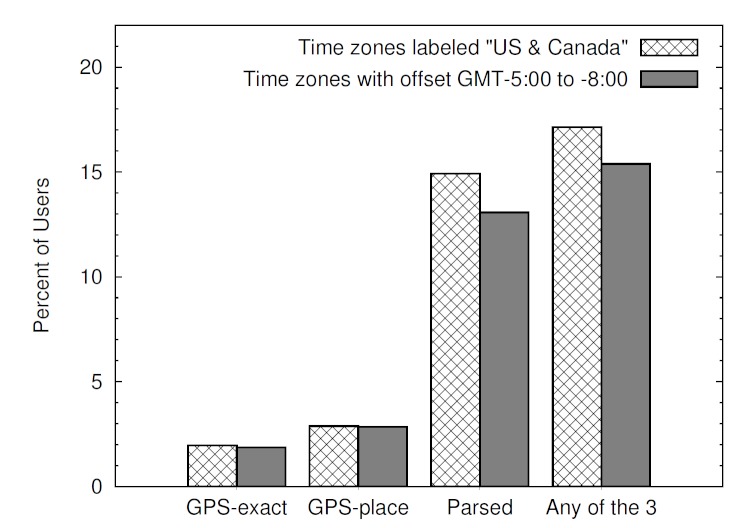
Twitter users providing location indicators in the US time zones.

Table 2 and Figure 2 show the number of users who matched our parsing criteria for the 4 continental US time zones, specifically Greenwich Mean Time (GMT) −5:00 to −8:00. When restricting to the US time zones, there is ambiguity about whether to include those that are specifically labeled as a US time zone, such as “Pacific Time (US & Canada)”, or simply those that contain a time zone offset that falls within the range of continental US time zones. For example, the time zone “Mexico City” is not labeled as a US time zone, yet its offset of GMT −6:00 is the same as Central Standard Time in the United States. Because the time zone may be automatically inferred by the user’s local time when creating an account, many users in the United States may have their time zone set to a different zone with the same offset. Thus, focusing on those specifically labeled as “US and Canada” is likely to miss some users, but focusing on those within the offset range is likely to include many Central and South American users. We have included results for both cases.

**Table 1:** Tweets and users providing location indicators.

Location indicator	Tweets		Users	
	n	%	n	%
Total (with and without location)	23,830,273	100	9,496,448	100
GPS-exact	216,900	0.91	140,451	1.48
GPS-place	458,295	1.92	241,010	2.54
GPS-exact or GPS-place	481,179	2.02	256,059	2.70
Time zone	18,347,947	76.99	6,831,414	71.94

**Table 2:** Location of Twitter users within the time zones of the United States.

Location indicator	Labeled “US & Canada”		Time zones GMT −5:00 to −8:00	
	n	%	n	%
Total (with and without location)	2,117,064	100	2,904,103	100
GPS-exact	41,416	1.96	53,997	1.86
GPS-place	60,979	2.88	82,322	2.83
Parsed state	315,819	14.92	379,576	13.07
Any (GPS-exact, GPS-place, or parsed state)	362,663	17.13	445,800	15.35

### Accuracy of User-Supplied Data

Users enter their location information in their user profiles themselves; thus, there is potential for inaccuracy. To evaluate the accuracy of the user-supplied profile location, we compared parsed state data and GPS coordinate data when both were available. City data may be too difficult to parse because individuals may live in one city and work or go to school in another. Therefore, a comparison of state data is more appropriate provided the same individuals are less likely to cross state boundaries repeatedly on a daily basis.

When GPS-exact data were available, we used the Yahoo! Place Finder API [31] to determine the state’s identity through a reverse GPS lookup service. When GPS-place data were available, we extracted the state name based on the Twitter Place Type (directly, when supplied, or using a reverse GPS lookup as described previously). We compared the state name obtained by these methods with the state name parsed from the user-supplied location information. Table 3 shows the results.

**Table 3:** Comparison of GPS location data to parsed location data.

GPS location indicator	State name parsed from user profile (n)	Matching parsed and GPS data	
		n	%
GPS-exact	16,009	13,935	87.04
GPS-place	21,092	18,599	88.18
Total	37,101	32,534	87.69

### Distribution in the United States

**Figure 3:**
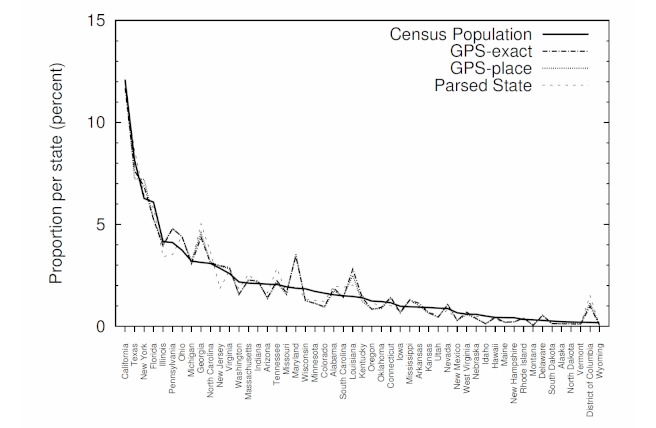
The proportion of Twitter users identified in each state and the proportion of the 2010 US census population in each state, ordered by census population.

With the parsing method in place, we extended our analysis of location information in Twitter to include parsed state data for the United States. Parsing international location data is a complex task, requiring such tools as standardization, place authority, and handling diverse conventions and languages. Figure 3 shows the proportions of users with parsed state data, with GPS-exact data and with GPS-place data in each state, and the proportions of 2010 US census population in each state. All of the location indicators correlate strongly (P < .001) with the population data (GPS-exact r = 0.97, GPS-place r = 0.97, and parsed r = 0.98).

**Figure 4:**
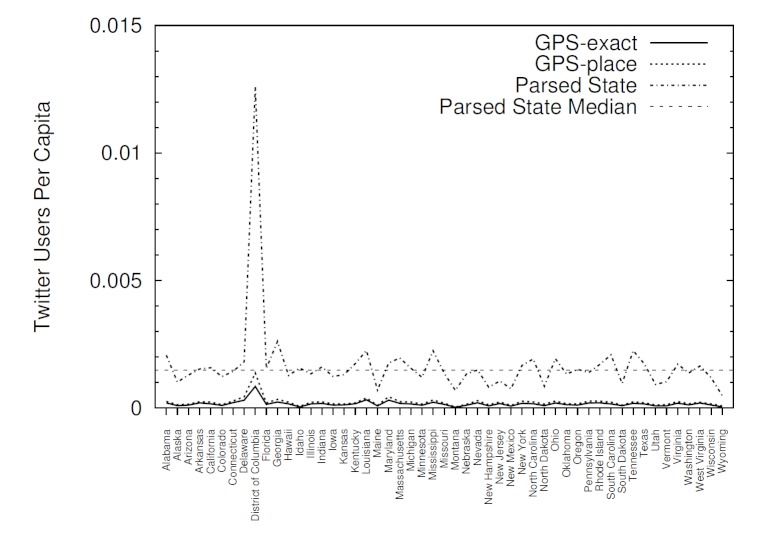
The number of geolocated Twitter users per capita in each state.

Figure 4 complements Figure 3 by showing the number of Twitter users in each state per capita (ie, divided by the census population) and the median value (0.0015) for the states identified through parsing. This does not represent the total number of registered Twitter users, but rather the number of unique users who posted during our sample period. The relatively high number of Twitter users in the District of Columbia, compared to its population is likely because users identify with and tweet from the metropolitan area, but actually reside in outlying suburbs in different states.

## Discussion

The purpose of this study was to document the prevalence of the location identification options available through Twitter and to present an estimate of the usability of each option. We have shown that there are several location indicators in Twitter and, when taken together, they offer a sizable sample of individuals whose location can be accurately inferred. This has clear implications for infoveillance, infodemiology, and “right time, right place” health communication.

Although only a small percentage of Twitter users provide reliable GPS coordinates (2.70%), there is actually a large number of users (and tweets) with GPS data because of the size of the overall data set. In the 2-week period of this study, the 2.02% of tweets that contained GPS information corresponded to 481,179 tweets. Because the sample is only 1% of the overall traffic on Twitter during those 2 weeks, if the same proportion were to hold true in the larger sample, we could infer that there were about 48 million tweets with GPS information posted during that period. With 2.5 tweets per user, this would correspond to approximately 19 million individuals. Furthermore, we saw that user-supplied location information matched GPS data in 87.69% of cases (in the United States). Hence, one could reliably use location information for between 15.35% and 17.13% of users. Interestingly, Keeter and colleagues compared the results of a 5-day survey employing the Pew Research Center’s methodology (with a 25% response rate) to those from a more rigorous survey conducted over a much longer field period and achieved a higher response rate of 50% [32]. In 77 of 84 comparisons, the 2 surveys yielded results that were statistically indistinguishable. Thus, it appears that surveys with lower response rates (20%) were only minimally less accurate. As a result, researchers can have additional confidence to value the location information available from Twitter, a real-time and real-place benefit of social media over traditional survey methodology.

Table 1 also shows that 2.70% of Twitter users broadcast their GPS location. Interestingly, this is a significantly lower figure than the 14% of social media users who use automatic location tagging on posts reported in a recent publication by the Pew Internet and American Life Project [33]. An obvious difference is that the Pew research considers all social media, whereas we have focused exclusively on Twitter. Additionally, some of the respondents represented in the Pew report could be using third-party location-tagging applications (eg, foursquare), which data may not appear in our sample, or they may be tweeting so infrequently that they would be underrepresented in our tweet-based sample. However, even considering these possibilities, the magnitude of the difference suggests that there may be additional factors. This difference warrants future research to determine the extent to which users are even aware that broadcasting GPS location is possible. It is plausible that users are largely unaware of such features or have minimal understanding with respect to how they function, both of which may attribute to this discrepancy.

An additional explanation for this (rather significant) discrepancy between 2.70% and 14% may be the distinct data collection approaches employed: questionnaires administered via phone interviews versus direct observation of user behavior on Twitter. Questionnaires can only report on what people perceive as opposed to what may actually be happening. For example, the question asked in the Pew questionnaire leading to the above result was “Thinking about the ways people might use social networking sites...Do you ever...Set up your account so that it automatically includes your location on your posts?” The answers included “Yes, do this: 14%” and “No, do not do this/have not done this: 84%.”

It is possible that respondents believed that GPS location was a default setting. This would lead to the conclusion that they had enabled location tagging for social media on their device, although GPS coordinates were not broadcast. From our own experience with the iPad 1, we found that the device itself may be GPS-enabled, yet the Twitter application on the device is not. Furthermore, the application could be GPS-enabled, yet coordinates are not broadcast because the location setting is not activated in the Twitter profile. In that sense, it is possible that someone may think that their tweets are location-tagged when, in fact, they are not. In this way, public health may benefit from eliciting additional location information that can be provided in the actual tweet. Twitter users who are otherwise willing to reveal their location, but are unaware of the default privacy settings, could be encouraged to provide such information. For example, followers of Twitcident (http://twitcident.com), a Dutch-based system for filtering emergency-related tweets, may feel inclined to tweet the location of emergency situations in an effort to assist emergency responders. Twitter prompts that ask users to tweet about their favorite locations to exercise may be useful in helping authorities allocate resources for promoting active lifestyles in areas where they are most likely to be successful.

As observed in this study, the parsed state data matched the GPS-derived state data 87.69% of the time. A mismatch does not necessarily mean that the user-supplied location was inaccurate or purposefully misleading, but it could represent a user tweeting from a business trip or vacation, or working in a metropolitan area across state lines. In this regard, the percentages in Table 3 are a lower bound and validate that the majority of the user-supplied locations are accurate for those users who provide GPS data and have profiles that can be parsed with our method. However, there is a potential bias in that users who are willing to broadcast their location might be more likely to tell the truth in their profile. Also, users who are unwilling to give an accurate profile location may be more likely to leave it empty or provide a non-descriptive location, as opposed to supplying an inaccurate, yet well-formed, location. This could be the focus of future research aimed at determining the extent to which Twitter users enable/disable GPS broadcasting and their reasons for doing so. For example, Twitter users vacationing in an exotic location may wish to enable GPS broadcasting, whereas others may disable broadcasting if their desire is to remain anonymous. This assumes that these users are aware of the toggle settings available for GPS broadcasting. Studies of this nature could establish the basis for determining the representativeness of GPS-enabled tweets. Moreover, this finding may question Twitter’s utility as a means for providing “right time, right place” tailored interventions, considering the location may not reflect the user’s actual setting, provided he or she knowingly deactivates location.

 As presented in this study, there is a significant level of consistency between the proportion of location-tagged tweets and state populations in the United States. This finding indicates that, at least within the United States, there is no evidence of disproportionate GPS enabling among states. Although much more information is needed to assess the true qualitative representativeness of Twitter (eg, ethnicity, age, and gender), this quantitative consistency is promising. Whereas it was beyond the purview of the current study to assess the validity of social media data, for public health researchers and communicators to dismiss such data sources without further consideration would be premature because it may miss an opportunity to observe, reach, and communicate with people in unprecedented ways. And although it is unlikely that social media could ever completely replace more traditional research methods (eg, questionnaires), it can certainly complement them and add a further dimension to research.

 In conclusion, we note that we have focused our attention on what users can do explicitly to specify their own location information. Although Twitter’s opt-in policy for location information is ethically sound, it would be interesting to study what could be done to encourage increased opt-in, for example, by working on dispelling concerns about how information is used or by demonstrating how information can be used for the good of all (eg, the Twitcident app). Furthermore, recent studies have demonstrated that it may be possible to infer location information based on either the words appearing in a user’s tweets [34] or the location of a user’s friends [35]. Further exploration of these ideas and other means of geographical prediction could augment the amount of location information available in social media.

## Abbreviations

API: application programming interface
app: application
GPS: Global Positioning System
GMT: Greenwich Mean Time

## References

[1] Pentland A, Lazer D, Brewer D, Heibeck T (2009). Using reality mining to improve public health and medicine. Stud Health Technol Inform.

[2] Estrin D, Sim I (2010). Health care delivery. Open mHealth architecture: an engine for health care innovation. Science.

[3] Eke PI (2011). Using social media for research and public health surveillance. J Dent Res.

[4] Farmer AD, Bruckner Holt CE, Cook MJ, Hearing SD (2009). Social networking sites: a novel portal for communication. Postgrad Med J.

[5] Jain SH (2009). Practicing medicine in the age of Facebook. N Engl J Med.

[6] Merchant RM, Elmer S, Lurie N (2011). Integrating social media into emergency-preparedness efforts. N Engl J Med.

[7] Chunara R, Andrews JR, Brownstein JS (2012). Social and news media enable estimation of epidemiological patterns early in the 2010 Haitian cholera outbreak. Am J Trop Med Hyg.

[8] Pandey A, Patni N, Singh M, Sood A, Singh G (2010). YouTube as a source of information on the H1N1 influenza pandemic. Am J Prev Med.

[9] Backinger CL, Pilsner AM, Augustson EM, Frydl A, Phillips T, Rowden J (2011). YouTube as a source of quitting smoking information. Tob Control.

[10] Burton S, Morris R, Hansen J, Dimond M, Giraud-Carrier C, West J, Hanson C, Barnes M (2012). Public health community mining on YouTube.

[11] Prier KW, Smith MS, Giraud-Carrier C, Hanson C (2011). Identifying health-related topics in Twitter: an exploration of tobacco-related tweets as a test topic.

[12] West JH, Hall PC, Prier K, Hanson CL, Giraud-Carrier C, Neeley ES, Barnes MD (2012). Temporal variability of problem drinking on Twitter. Open Journal of Preventive Medicine.

[13] Heaivilin N, Gerbert B, Page JE, Gibbs JL (2011). Public health surveillance of dental pain via Twitter. J Dent Res.

[14] West J, Hall PC, Hanson C, Thackeray R, Barnes M, Neiger B, McIntyre E (2011). Breastfeeding and blogging: Exploring the utility of blogs to promote breastfeeding. Am J Health Educ.

[15] Paul MJ, Dredze M (2011). You are what you tweet: analyzing Twitter for public health.

[16] Scanfeld D, Scanfeld V, Larson EL (2010). Dissemination of health information through social networks: Twitter and antibiotics. Am J Infect Control.

[17] Eysenbach G (2009). Infodemiology and infoveillance: framework for an emerging set of public health informatics methods to analyze search, communication and publication behavior on the Internet. J Med Internet Res.

[18] Ratzan SC (2011). Our new "social" communication age in health. J Health Commun.

[19] Gibbons MC, Fleisher L, Slamon RE, Bass S, Kandadai V, Beck JR (2011). Exploring the potential of Web 2.0 to address health disparities. J Health Commun.

[20] Adair JG (1984). The Hawthorne effect: a reconsideration of the methodological artifact. J Appl Psychol.

[21] Brownstein JS, Freifeld CC, Madoff LC (2009). Digital disease detection--harnessing the Web for public health surveillance. N Engl J Med.

[22] Kreuter MW, Strecher VJ, Glassman B (1999). One size does not fit all: the case for tailoring print materials. Ann Behav Med.

[23] Strecher V, Wang C, Derry H, Wildenhaus K, Johnson C (2002). Tailored interventions for multiple risk behaviors. Health Educ Res.

[24] Strecher VJ, McClure JB, Alexander GL, Chakraborty B, Nair VN, Konkel JM, Greene SM, Collins LM, Carlier CC, Wiese CJ, Little RJ, Pomerleau CS, Pomerleau OF (2008). Web-based smoking-cessation programs: results of a randomized trial. Am J Prev Med.

[25] Patrick K, Intille SS, Zabinski MF (2005). An ecological framework for cancer communication: implications for research. J Med Internet Res.

[26] Heron KE, Smyth JM (2010). Ecological momentary interventions: incorporating mobile technology into psychosocial and health behaviour treatments. Br J Health Psychol.

[27] Intille SS, Kukla C, Farzanfar R, Bakr W (2003). Just-in-time technology to encourage incremental, dietary behavior change. AMIA Annu Symp Proc.

[28] Lampos V, Cristianini N (2012). Nowcasting events from the social web with statistical learning. ACM TIST.

[29] Smith A, Brenner J (2012). Twitter use 2012.

[30] (2011). Twitter Blog.

[31] (2011). Yahoo! Developer Network.

[32] Keeter S, Kennedy C, Dimock M, Best J, Craighill P (2006). Gauging the impact of growing nonresponse on estimates from a national RDD telephone survey. Public Opin Q.

[33] Zickuhr K, Smith A (2011). 28% of American adults use mobile and social location-based services.

[34] Cheng Z, Caverlee J, Lee K (2010). You are where you tweet: a content-based approach to geo-locating Twitter users.

[35] Backstrom L, Sun E, Marlow C (2010). Find me if you can: improving geographical prediction with social and spatial proximity.

